# Integration of Facial Scanning Technology for Full‐Arch Implant Rehabilitation and Fabrication of Magnet‐Based Surgical Guide and Provisional Prosthesis: A Case Report

**DOI:** 10.1155/crid/5495934

**Published:** 2026-02-25

**Authors:** Mohamed Gebril, Faraj Edher

**Affiliations:** ^1^ Department of Oral Health Sciences, Faculty of Dentistry, University of British Columbia, Vancouver, British Columbia, Canada, ubc.ca

## Abstract

**Introduction and Objectives:**

Digital dentistry applications have been widely used in different aspects of prosthodontics. Multiple studies in the literature report the application of different technologies. The aim of this study was to focus on the combination of facial scanning, intraoral scanning, and cone‐beam radiographs for optimal implant planning and prosthodontic rehabilitation.

**Methods:**

Complex prosthodontic treatment of maxillary and mandibular implant rehabilitation utilizing the application of a novel technique of digital technology was explored. In this case report, facial scan, intraoral scan (IOS), and cone‐beam computed tomography (CBCT) images were merged for the virtual planning of implant placement.

**Results:**

A stackable magnet‐based surgical guide was fabricated and utilized for implant placement. Furthermore, a provisional implant‐supported prosthesis was prefabricated for immediate loading and chairside pickup.

**Conclusion:**

As the field continues to evolve, new technological advances offer more precise, predictable, and efficient implant rehabilitation.

**Clinical Significance:**

This technique is poised to become a component in the practice of advanced prosthodontics, adding to the landscape of dental implantology with its precision, predictability, and patient‐centered focus.

## 1. Introduction

Full‐arch implant rehabilitation presents one of the challenging clinical scenarios in prosthodontics, making accurate analysis across multiple criteria essential to achieve predictable outcomes. Criteria include the smile line, prosthetic space, material selection, patient preferences, bone availability, patient dexterity, and prosthesis design (removable vs. fixed) [1–4].

A prosthetically driven treatment planning approach, where implant placement aligns with a prosthetic plan, significantly enhances final outcomes. In the last decade, we have witnessed substantial advancements in digital technology in implant dentistry, allowing clinicians to improve predictability and efficacy in their implant rehabilitations by incorporating intraoral scanners, cone‐beam computed tomography (CBCT), facial scanners, and implant planning software [4–6].

Integrating these digital files allows clinicians to accurately design precise surgical guides based on a virtual prosthetic plan, mimicking the final design [4–7]. This clinical report introduces a technique for the planning and the fabrication of a magnet‐based surgical guide for full‐mouth implant rehabilitation. This includes a description of the integration of 3D facial scanning in the planning and an outline of the execution process.

## 2. Clinical Report

A medically fit female patient sought prosthodontic consultation, and upon clinical and radiographic assessment, we noted mobility of several teeth, defective restorations, secondary caries, high caries risk, and multiple inadequate root canal treatments. The decision to proceed with a maxillary and mandibular full‐arch implant rehabilitation was made in conjunction with the patient due to the compromised condition of the existing teeth and with consideration of the poor long‐term prognosis of the remaining dentition. Records were then initiated, including a full clinical photo series, CBCT scan, 3D facial scan (RAYFACE 3D Face Scanner‐RFS200), and intraoral scan (IOS).

**Figure 1:**
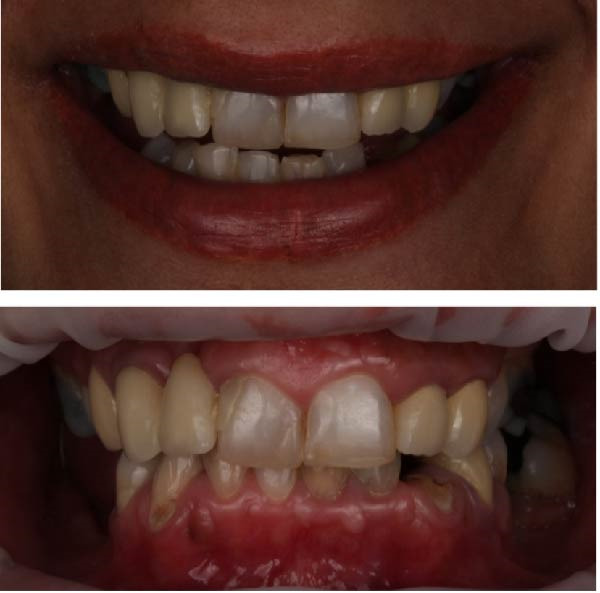
Initial clinical presentation (highest smile line and intraoral clinical photo).

**Figure 2:**
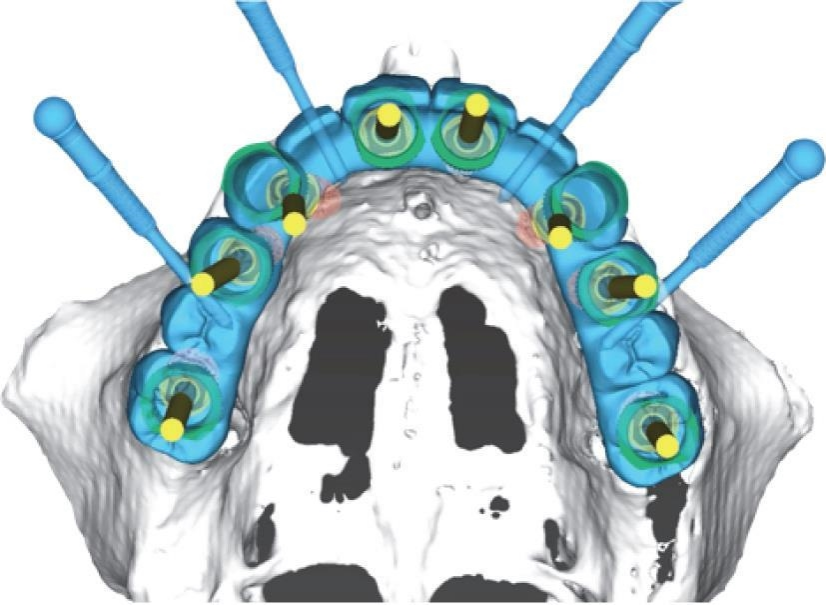
Virtual plan for maxillary implant placement illustrating prosthetically driven implant positioning based on CBCT data and the planned definitive restoration.

**Figure 3:**
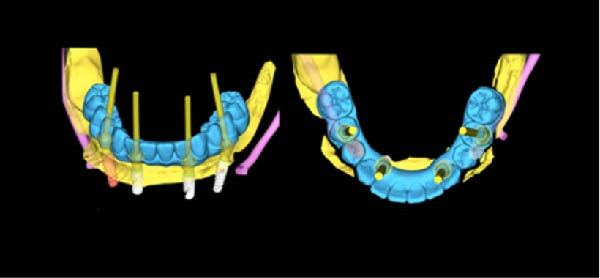
Virtual plan for mandibular implant placement illustrating prosthetically driven implant positioning based on CBCT data and the planned definitive restoration.

**Figure 4:**
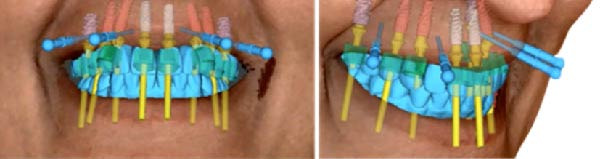
Integration of the facial scan with the virtual implant plan demonstrating alignment of implant positioning with facial landmarks, esthetic parameters, and prosthetically driven restorative planning.

Merging these data in implant planning software, as shown in Figures 1–4, facilitated a comprehensive assessment of the patient. The 3D facial scan guided implant planning, incorporating the assessment of lip support, smile line, and buccal corridors. The implant planning software utilized in this case was coDiagnostiX (Dental Wings GmbH, Chemnitz, Germany).

The treatment plan was determined after exploring all the possible options, including fixed and removable implant rehabilitation options. The plan involved an FP1 maxillary prosthesis and an FP3 mandibular prosthesis. The merged data facilitated the fabrication of a complete full‐mouth wax‐up, addressing incisal edge positions and gingival margin positions on the maxillary teeth to achieve the ideal proportions, lengths, incisal plane, curve of Spee, and buccal corridor. This design ensured an optimal setup for the mandibular anterior teeth to achieve 2 mm of overjet/overbite and distributed occlusal contacts on all teeth in maximum intercuspal position (MIP) by more naturally following a similar morphology to the existing teeth with more ideal proportions, positions, line angles, and contours.

**Figure 5:**
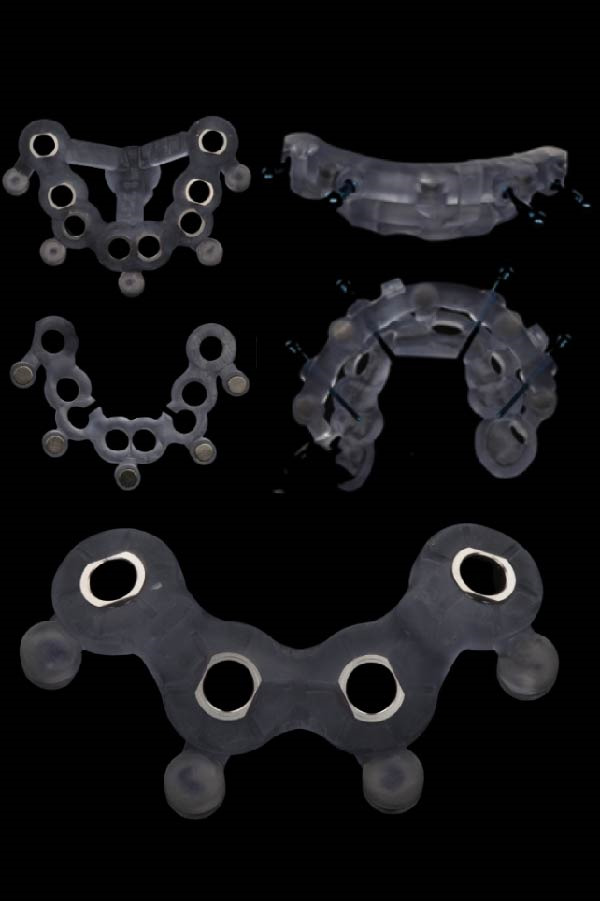
The maxillary and the mandibular surgical guide, fixation pin guide, and SRA implant placement guide for the maxillary arch.

**Figure 6:**
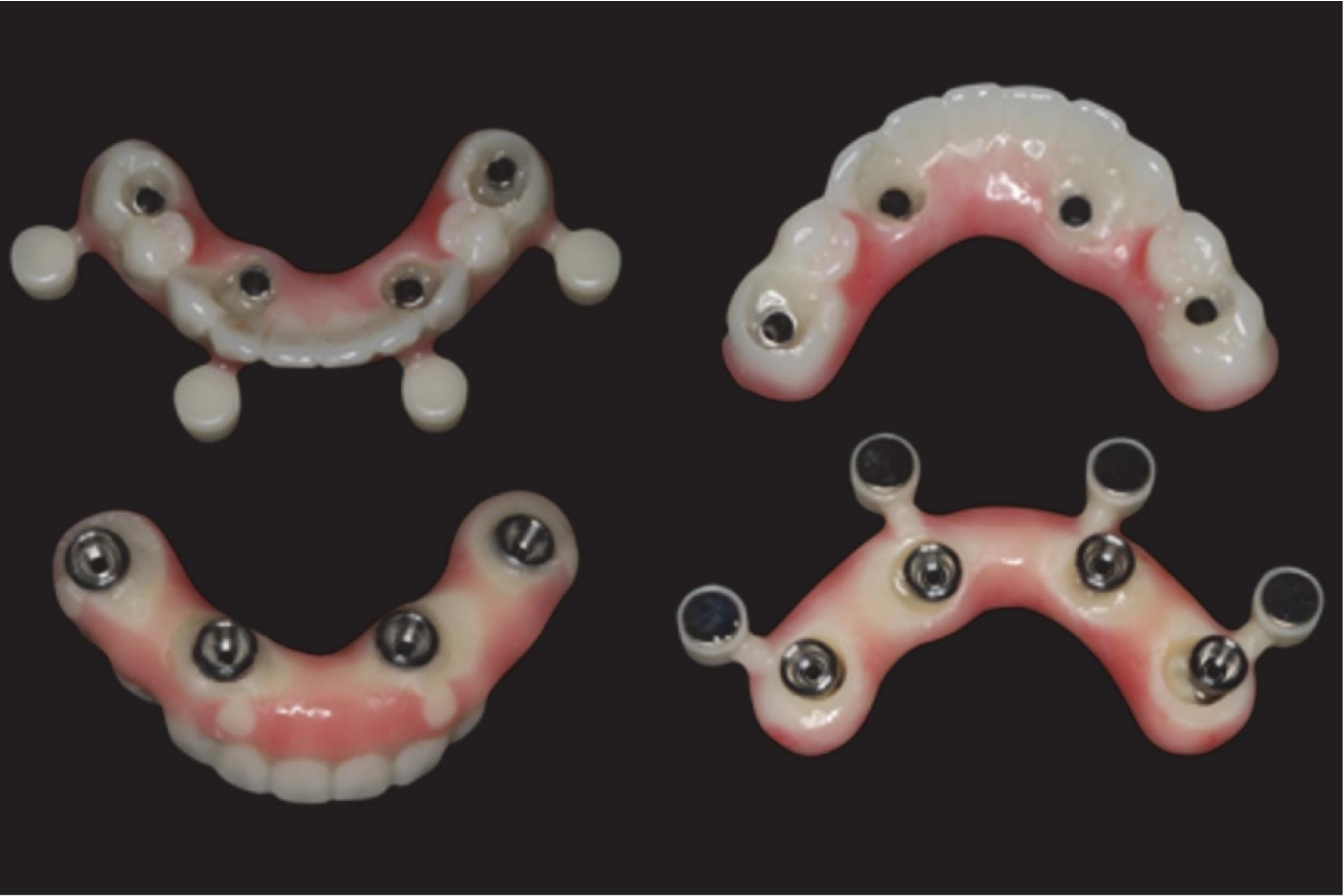
The chairside picked up a provisional mandibular fixed prosthesis with the magnet‐based seating guide.

**Figure 7:**
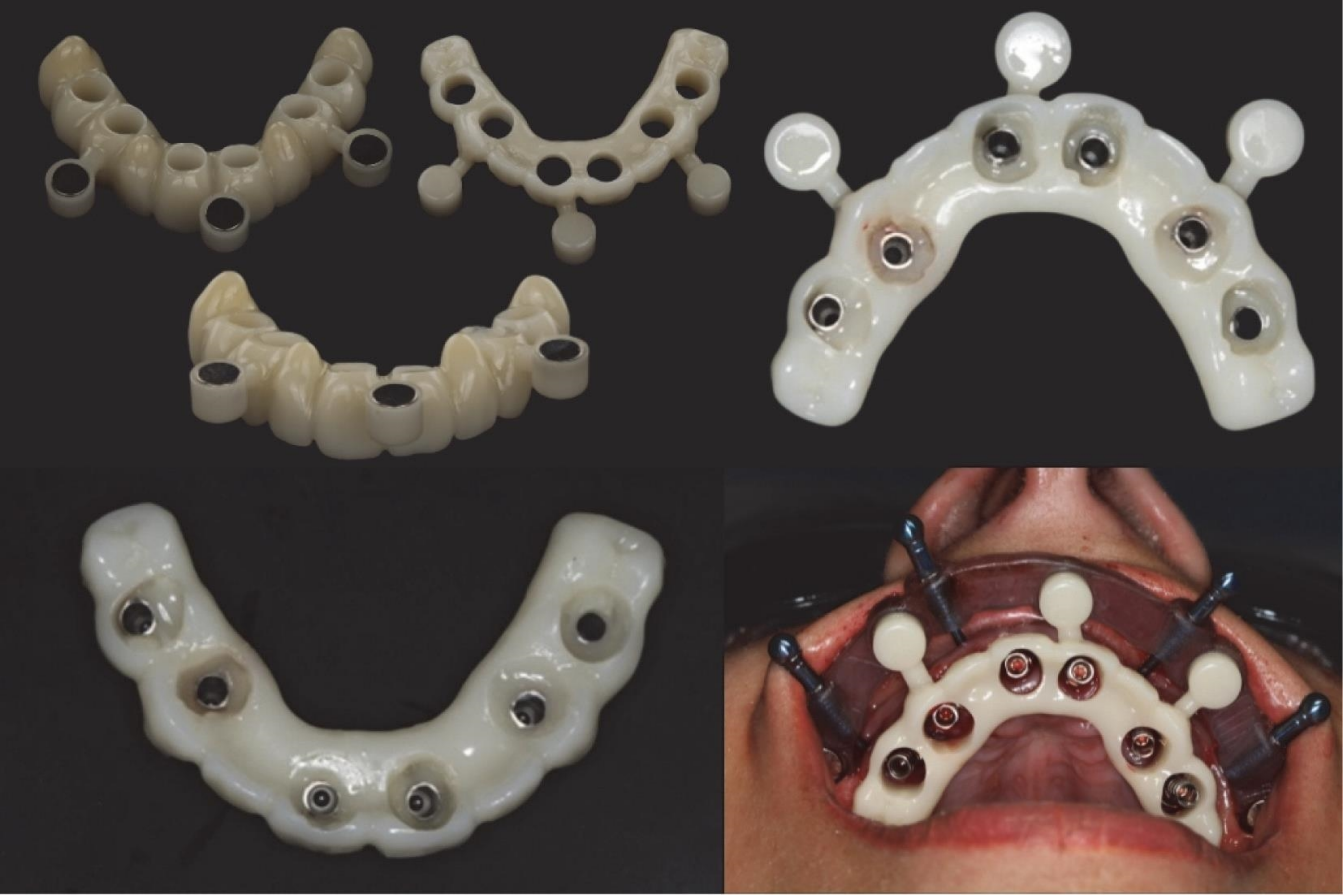
The chairside picked up a provisional maxillary fixed prosthesis with the magnet‐based seating guide.

Based on the ideal prosthetic plan (as described), the implant positions were planned, accounting for anatomy, anterior–posterior spread, and distribution of the implants. Both surgical and prosthetic plans were reviewed and approved, and magnet‐based surgical guides were fabricated (Figures 5 and 6) to ensure predictable seating of the guide components onto a base guide affixed utilizing fixation pins. This allowed the interchangeable placement and removal of the surgical guide for implant placement and the prefabricated polymethyl methacrylate (PMMA) provisional for chairside pickup of the immediate prosthesis. Eight maxillary and four mandibular implants were placed using the Straumann implant system with an RC prosthetic platform (Straumann, Basel, Switzerland). The prosthesis, a PMMA Provisional version from Global Laboratories, included prefabricated holes for passive fitting over temporary cylinders (with 2 mm of circumferential space), as shown in Figure 7. The chairside pickup was done with flowable composite around the temporary cylinders. Afterwards, both prostheses were fully contoured and polished, only requiring finishing around the temporary cylinders after chairside pickup. Implant placement adhered to the planned scenarios using fully guided protocols, and immediate loading with a chairside provisional prosthesis was achieved using the magnet‐based guide for the chairside pickup using flowable composite. Initial primary stability was achieved and confirmed to allow for immediate loading. Insertion torque reached 35 Ncm, confirming adequate primary stability. For the maxillary arch, there was no plan for any bone reduction considering the FP1 design of the prosthesis. However, for the mandibular arch, bone reduction was incorporated to allow for proper depth of implant placement and prosthetic space creation. The provisional prosthesis was contoured, polished, and radiographically verified to assure passive fit. The FP1 design was implemented for the maxillary arch for a more natural appearance and also to avoid unnecessary bone reduction. On the other side, the mandibular arch design and position of the teeth detected more bone reduction where white and pink replacement was needed. For that reason, the FP3 design was adopted in the mandible.

## 3. Discussion

The advent of guided protocols in full‐arch implant rehabilitation marks a transformative shift in the planning and execution of these procedures [5, 8, 9]. While traditional methods have been effective, the precision and predictability offered by modern guided techniques allow for many added benefits [3, 9]. These systems, evolving from the earlier cumbersome stackable guides, now enable simultaneous surgical and prosthetic guidance, closely adhering to the preplanned prosthetic design [10, 11].

A significant advancement in these protocols is the integration of 3D facial scanning with implant planning. This technology allows for the incorporation of diagnostic elements and variables that two‐dimensional imaging cannot effectively provide [12]. By doing so, it enhances the overall predictability and accuracy of the restorative plan, ensuring that the final outcome is uniquely designed to the individual’s facial characteristics [13, 14]. However, these advanced techniques and technologies are not without limitations. The critical assessment of differences in every clinical scenario and the implementation of this workflow must be considered. The additional time and costs associated with planning, the need for proficiency in multiple software platforms for planning, designing, and fabricating guides and temporaries, and the requirement for familiarity with guided surgical protocols can be considerable drawbacks [13–17].

These factors can pose challenges, particularly in settings where resources are limited or where practitioners may not have extensive experience with these technologies [14–18]. Additionally, while these systems offer substantial benefits, their complexity and the need for precise execution mean that there is a learning curve and an ongoing need for professional development [18, 19]. The authenticity in the technique presented in this case report is that it can be applied with different clinical presentations that allow for proper articulation. The fact that the patient in this report had multiple remaining teeth allowed more predictable articulation using the patient’s dentition. This is an introduction of a new method of merging different aspects of the technology for full‐arch fixed implant rehabilitation. The utilization of the patient’s dentition as a critical landmark helped in planning for the surgical execution and the immediate temporization. However, in the case of an edentulous patient, a well‐made complete denture is essential to allow for using this technique predictability.

As with any advanced technology, there is also the potential for technical issues or inaccuracies, which can impact treatment outcomes [3, 14, 19]. Moreover, the increased cost and the overall time needed for planning can also be considered additional limitations of this workflow. Despite these limitations, the future of implant dentistry lies in the further development and adoption of these technologies. The field is expected to continue evolving, with advancements in both software and materials. This evolution will likely lead to even more refined and user‐friendly systems, further driving the adoption of these techniques in clinical practice.

## 4. Summary

The case reported described the integration of 3D facial scanning and the fabrication of a magnet‐based stackable guided system in full‐arch implant rehabilitation. The application of this technique, as demonstrated in the clinical report, allowed precise planning and execution. The use of 3D facial scanning in particular brings a new dimension to implant dentistry, allowing for a more comprehensive and patient‐specific approach. It addresses nuances in facial anatomy and smile esthetics that traditional two‐dimensional imaging cannot capture, thereby elevating the standard of care in prosthodontics. This report represents one clinical scenario, and variations in clinical situations should be considered before applying this technique. With critical analysis of potential limitations and considerations for different clinical case presentations, this technique is poised to become a component in the practice of advanced prosthodontics, adding to the landscape of dental implantology with its precision, predictability, and patient‐centered focus.

## Funding

No funding was received for this manuscript.

## Consent

Informed patient consent has been obtained.

## Conflicts of Interest

The authors declare no conflicts of interest.

## Data Availability

The data that support the findings of this study are available upon request from the corresponding author. The data are not publicly available due to privacy or ethical restrictions.
